# Extracellular vesicles: novel vehicles in herpesvirus infection

**DOI:** 10.1007/s12250-017-4073-9

**Published:** 2017-10-30

**Authors:** Lingzhi Liu, Quan Zhou, Yan Xie, Lielian Zuo, Fanxiu Zhu, Jianhong Lu

**Affiliations:** 1 The Key Laboratory of Carcinogenesis of the Chinese Ministry of Health, Xiangya Hospital, Central South University, Changsha, 410080 China; 2 Cancer Research Institute, Central South University, Changsha, 410078 China; 3 Faculty of Chemical, Environmental and Biological Science and Technology, Dalian University of Technology, Dalian, 116024 China; 4 Department of Microbiology, School of Basic Medical Science, Central South University, Changsha, 410078 China; 5 Department of Biological Science, Florida State University, Tallahassee, 32306 USA

**Keywords:** herpesviruses, extracellular vesicles (EVs), infection, pathogenesis

## Abstract

Herpesviruses are remarkable pathogens that have evolved multiple mechanisms to evade host immunity, ensuring their proliferation and egress. Among these mechanisms, herpesviruses utilize elaborate extracellular vesicles, including exosomes, for the intricate interplay between infected host and recipient cells. Herpesviruses incorporate genome expression products and direct cellular products into exosomal cargoes. These components alter the content and function of exosomes released from donor cells, thus affecting the downstream signalings of recipient cells. In this way, herpesviruses hijack exosomal pathways to ensure their survival and persistence, and exosomes are emerging as critical mediators for virus infection-associated intercellular communication and microenvironment alteration. In this review, the function and effects of exosomes in herpesvirus infection will be discussed, so that we will have a better understanding about the pathogenesis of herpesviruses. 
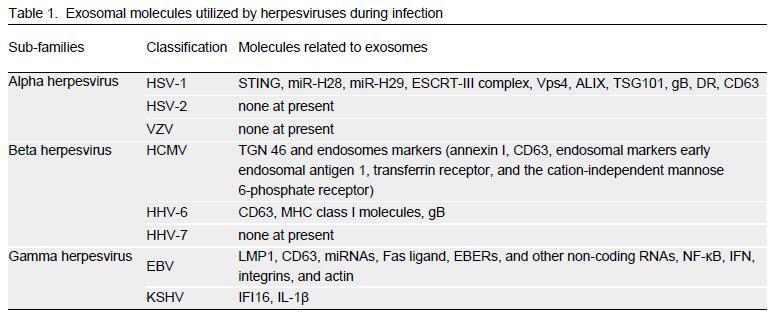

## INTRODUCTION

Human herpesviruses (HHVs) are distributed worldwide, and more than 90% of adults are infected by one or multiple HHVs (van Diemen and Lebbink, 2016). Once the host is attacked by these viruses and incapable of eliminating the pathogen, HHVs can establish lifelong latent infections and cause symptoms, even resulting in serious diseases such as shingles and tumors (van Diemen and Lebbink, 2016; Olsson et al., 2017). The HHV family contains three contagious sub-families: the alpha herpesvirus family [herpes simplex virus 1(HSV-1), HSV-2, and varicella-zoster virus (VZV)], beta herpesvirus family [human cytomegalovirus (HCMV), HHV6, and HHV7], and gamma herpesvirus family [Epstein-Barr virus (EBV) and Kaposi's sarcoma–associated herpesvirus (KSHV)] (Dreyfus, 2013). A seroepidemiology of HHVs was conducted using 3, 444 follow-up samples, and the results revealed that the yearly incidence of HHVs remains high (Olsson et al., 2017). Among the HHV family members, EBV and KSHV have been confirmed to be tumor viruses.

Extracellular vesicles (EVs) have recently been revealed as itinerant cargo that engage in intercellular communication and immunity regulation (Szatanek et al., 2017). There are different types of EVs, including exosomes, apoptotic bodies, microvesicles, and retrovirus-like particles (Akers et al., 2013). Because exosomes are the most widely studied type of EVs, the characterization of exosomes will be presented in more detail in this review.

Since the first evidence of EVs was presented, there have been many studies on the origin and biogenesis of EVs, in which the endosomal network is quite essential. Vesicles that are destined to form exosomes are fused with early endosomes and then shed into late endosomes [also termed multivesicular bodies (MVBs)]. During this process, endosomes sort their contents into vesicles, and late endosomes then fuse with the plasma membrane, followed by their excretion out of the cell to become exosomes (Akers et al., 2013). A Golgi component in exosomes has been confirmed (Sotelo and Porter, 1959; Lee Y et al., 2012). Although the precise mechanisms regarding the origins of exosomes remain unclear, it has been claimed that lipid rafts (which form divided microdomains), transmembrane proteins, protein complexes such as endosomal sorting complexes required for transport (ESCRT), and Rab proteins (which modulate vesicular traffic) are responsible for exosome biogenesis (Duijvesz et al., 2011; Hurley, 2015). However, conclusive reports to confirm the exact process for shaping exosomes are lacking.

Exosomes are nanoscale EVs and play critical roles in cell-to-cell communication. Exosomes contain DNA, RNA, proteins, and other bioactive molecules, allowing the exchange of genetic information and regulation of related physiological and pathological activities. Numerous studies have shown that exosomes released from tumor cells may affect tumor formation, growth, angiogenesis, metastasis, and drug resistance. Furthermore, exosomes assist tumor cells in avoiding immune surveillance and prevent the tumor niche generation (Zhang et al., 2015b; Tkach and Théry, 2016). In addition, aside from their role in tumor progression and metastasis, exosomes can protect against bacterial and viral infection, adjust tumor immunity, and mediate the immune suppression of host cells (Wang et al., 2017). Exosomes have become a popular research topic, resulting in intensive basic or clinical studies on their roles in tumor development and use as targets for drugs.

Even though the immune system employs various methods to fight and remove pathogens, viruses always evolve multiple counteracting mechanisms to evade the host immune system and allow their persistence (Anderson et al., 2016). The emerging data on microvesicles, including exosomes, represent a potent weapon in efforts to protect against viruses. When infecting host cells, viruses inject their own components into exosomes. Once released, exosomes convey their contents, affecting neighboring and distant recipient cells. Cumulatively, viral nucleic acids, such as non-coding RNAs, and proteins expressed in exosomes deceive the immune system and even influence uninfected host cells, allowing viruses to achieve permanent and even lifelong persistence (Anderson et al., 2016; Hancock and Skalsky, 2017). Recently, David M Knipe and his colleagues summarized some viral gene products that can promote the epigenetic silencing of genomes, placing great emphasis on their potential as a promising target in latent infections (Knipe et al., 2017). Similarly, HHVs can also utilize these important exosomal vehicles to mediate their pathological process and facilitate cell-cell communication. In this review, we will provide an update on the function of exosomes in HHV infections, with the aim of advancing the knowledge on the pathogenesis and persistence of herpesvirus infection and providing novel insights into virus-host interactions.

## EXOSOMES IN ALPHA HERPESVIRUS INFECTION

Alpha herpesviruses include important pathogens, such as HSV-1, HSV-2, and VZV (Hogue et al., 2016). Increasing lines of evidence indicate that alpha herpesviruses utilize exosomes to deliver cargo such as transcripts (mRNAs, miRNAs, and DNA) and proteins.

HSV-1 infects the majority of the world's population at some point in their lives. The innate immune system, including macrophages, responds to the viral attack. A research team (Miettinen et al., 2012) identified and analyzed 516 human proteins from the secretome of HSV-1-infected macrophages and found that a large proportion of the secreted proteins were intracellular proteins and nearly 80% of the proteins whose secretion increased more than 2-fold were known exosomal proteins. Moreover, their study also revealed that HSV-1-infected macrophages possibly play a role in antiviral defense, thus providing a global perspective of HSV-1. In addition, the stimulator of IFN genes (STING), a vital innate immune adaptor to HSV-1 infection, plays a critical role in the course of infection. A report demonstrated that STING as well as viral miRNAs and mRNAs are transported via exosomes from infected cells (Kalamvoki M et al., 2014). While some viral mRNAs may silence viral genes in latent infected cells, this report suggested that HSV-1 controls its virulence to ensure dissemination between individuals. This might represent a strategy used by the virus to avoid being eliminated by host cells and establish lifelong infection in balance with the host (Kalamvoki Maria and Deschamps, 2016). According to another report, two HSV-1 miRNAs termed miR-H28 and miR-H29 control viral replication and transmission from infected to uninfected cells through ectopic expression before infection. Because HSV-1 is transmitted by contact between transmitters and recipients, viral replication and transmission are requirements for the effective spread of the virus (Han et al., 2016).

It is well recognized that the envelopment and egress of viruses are complicated processes, and HSV-1 forms mature viruses through the budding of viral glycoproteins and nucleocapsids into the lumens of cytoplasmic membranous compartments. HSV-1 was the first large assembled DNA virus to be reported as being sorted into MVBs (Crump et al., 2007). Vps4, an enzyme required for producing luminal vesicles, is essential for the cytoplasmic assembly of HSV-1. Moreover, Vps4 is also an ATPase that is required for four protein complexes that function as ESCRTs. It has been demonstrated that the dominant-negative ESCRT-III complex overwhelmingly inhibits HSV-1 replication by preventing virus envelopment, in line with the role of Vps4. The ESCRT-III complex and Vps4 are packed into mature HSV-1 virions and exosomes, whereas ALIX and TSG101, two dominant-negative proteins, are detectable at low levels and contribute to HSV-1 production (Pawliczek and Crump, 2009). Specifically, for many enveloped viruses, ESCRT provides sites for viral proteins involved in assembly and envelopment to interact intimately, whereas viruses, including HSV-1, recruit ESCRT components to specific compartments. In addition, glycoprotein B (gB, encoded by HSV-1), HLA-DR (DR) molecules, and CD63 (a late endosomal marker) are also present in exosomes. The glycoprotein of gB manipulates DR via hijacking DR from the primary transport route into the exosomal pathway and thus provides a new method of viral immune evasion (Temme et al., 2010). Moreover, the site of intracellular gB varies, indicating that gB is dependent on the exosomal pathway. The intracellular trafficking of gB and the envelopment/egress of HSV-1 rely on MVB biogenesis (Calistri et al., 2007). This further suggests that the sorting of gB into MVB membranes is obligatory for the formation of mature HSV-1 and that modified MVB membranes provide a platform for the recruitment of MVB components to allow virus envelopment and egress.

HSV-2 is one of the most prevalent genital pathogens in humans, infecting the genital tract mucosa (Lee and Ashkar, 2012; Zhu et al., 2017). HSV-2 is also involved in HIV infection and prevalence, and the ability of HSV-2 to evade the immune response and establish a latent infection has made it difficult to develop an effective vaccine (Chan et al., 2011). The innate and adaptive immune processes underlying the response to HSV-2 provide a powerful foundation for the research and development of an effective HSV-2 vaccine (Akinyi et al., 2017; Zhu et al., 2017). However, to date, there has not been a study on the relationship between HSV-2 infection and exosomes.

VZV is a highly contagious virus that only affects humans. VZV can cause chickenpox among the elderly and immunocompromised individuals during acute infection (Ahmed et al., 2015). Although primary chickenpox is typically self-limited, VZV can remain dormant in the nervous system and then is reactivated in a weak host, leading to shingles or inflammation (Kurapati et al., 2017). Given that T cells play a crucial role in disseminating VZV to the skin and ganglia in the early period of VZV infection, a nonhuman primate model of VZV infection was used to demonstrate VZV-T cell interactions. The data revealed that the gene expression of the model for regulating cell metabolism, antiviral immunity, and the cell cycle were altered (Arnold and Messaoudi, 2017). To date, no study has addressed the role of exosomes in VZV.

## EXOSOMES IN BETA HERPESVIRUS INFECTION

The beta herpesvirus family contains HCMV, HHV6, and HHV7. HCMV has been widely studied; however, data on HHV6 and HHV7 remain scarce.

HCMV is a prototypical beta herpesvirus. With nearly 40 known gene products, HCMV is involved in multiple physical and pathological processes, including processing and presenting antigens, interferon and chemokine signaling, initiating apoptotic signaling, and functioning as a cytokine, to evade and subvert the host immune defense (Hudson, 2014). HCMV infection is always associated with acute allograft ejection and chronic allograft vasculopathy in transplant patients whose immune system is suppressed. By stimulating endothelial cells (ECs) to release antigenic exosomes, HCMV exacerbates allograft rejection in response to the allogeneic $CD4^+$ memory T cell-mediated immunity (Walker et al., 2009), revealing a novel manner for EC-derived exosomes to evade immune surveillance. In addition, because ESCRT machinery is important for incorporating the viral cargo into MVBs, HCMV is reported to undergo maturation independent of ESCRT components, different from HSV-1 (Fraile-Ramos et al., 2007). However, another study reported that the final step in HCMV production requires the ESCRT machinery (Tandon et al., 2009). Thus, the exact site of the final envelopment for HCMV remains unclear and warrants further investigation. Based on the study by Cepeda V *et al.*, HCMV generates vesicles that traffic between the trans-Golgi network (TGN) and endosomes. The vesicles include the TGN and endosome markers (TGN46, annexin I, CD63, endosomal marker early endosome antigen 1, transferrin receptor, and the cation-independent mannose 6-phosphate receptor) to achieve viral envelopment (Cepeda et al., 2010). Nevertheless, the mechanisms involved in virus envelopment and egress still await detailed elucidation.

Closely related to HCMV, HHV-6 is a notable beta herpesvirus and can cause primary infection or be activated from latency in liver transplant recipients (Parra et al., 2017). HHV-6 is divided into two distinct species:

HHV-6A and HHV-6B. Markedly different from other HHVs, HHV-6 integrates its genomic DNA into the subtelomeric part of cell chromosomes in approximately 1% of the population (Agut et al., 2015). Evidence of its exosomal presence was presented in a study by Yasuko Mori *et al*. The authors showed that cells that were infected with HHV-6 were larger than uninfected ones and contained MVBs in the late period of infection. As noted above, exosomes are derived from MVBs and eventually fuse with the plasma membrane. MVBs contain CD63, which is an MVB late endosome marker and envelope glycoprotein, that is localized in internal vesicles. The available evidence indicates that HHV-6 virions are released through the exosomal pathway, which demonstrates the significance of exosomes in the HHV6 maturation pathway (Mori et al., 2008). Additionally, MHC class I molecules are another rare member that have been found to be inserted into viral particles and exosomes and then released into the extracellular space via an exosomal secretary pathway, which can be blocked by the U24 protein of HHV-6 (Sullivan and Coscoy, 2010; Ota et al., 2014). Because HHV-6 mainly replicates in T lymphocytes, MHC class I molecules are colocalized with the gB protein and incorporated into exosomes and modify HHV-6 infection (Ota et al., 2014). However, additional details about the role of exosomes in HHV-6 infection need to be addressed in future studies.

HHV-7 infection commonly occurs in infants or during childhood and may cause symptoms in cases of immunodeficiency after reactivation from a latent infection (Parra et al., 2017; Riva et al., 2017). Nevertheless, the pathogenic role of HHV-7 for non-immunosuppressed adults in diseases of the central nervous system still needs to be explored, and HHV-7 is possibly an etiological agent of suspected infectious encephalitis in individuals who do not respond to acyclovir (Parra et al., 2017). There is no published report on the roles of exosomes in HHV-7 infection.

## EXOSOMES IN GAMMA HERPESVIRUS INFECTION

The gamma herpesvirus family is made up of EBV and KSHV, both of which are closely associated with multitudinous malignancies. Even though EBV and KSHV were originally observed in human tumor specimens in 1964 and 1994, respectively, the finding that a portion of the human population was infected by both EBV and KSHV received great attention (Pegtel, 2013). When the two herpesviruses combine in aggressive lymphoma, primary effusion lymphoma (PEL), they can bring about an extraordinary synergic effect on lymphomagenesis, leading to increased complications (Meckes et al., 2013; Choi et al., 2017). A large number of investigations have revealed that these two viruses share similar colonization and persistence strategies, such as deregulating NF-κB to control B-cell proliferation and secreting proinflammatory mediators that contribute to tumorigenesis. Exosomes are another key strategy for the virus-associated tumor cells to facilitate the development of an adaptive tumor microenvironment that shapes the tumor (Pegtel, 2013). To confirm the effects of EBV and KSHV on exosomal content, proteomics techniques, including mass spectrometry, were performed on exosomes derived from cell lines that were uninfected, infected with alternative, or infected with both EBV and KSHV. The results indicated that exosomes from cells infected by these two viruses probably regulate cell death and survival, protein synthesis, ribosome function, and some signaling pathways. EBV-related exosomes mainly influence cellular signalings such as NF-κB, IFN, integrins, and actin, whereas KSHV-related exosomes prevailingly impact cell metabolism (Meckes et al., 2013). Gamma herpesviruses alter metabolic phenotypes to allow viral infection and everlasting persistence, and Angela Kwok-Fung Lo *et al*. speculated that this modulation is conductive to tumor progression, suggesting a new opportunity for therapeutic intervention (Lo et al., 2017).

EBV was the first human tumor virus confirmed to be associated with diverse malignancies, including nasopharyngeal carcinoma (NPC), Hodgkin's lymphoma, Burkitt's lymphoma, and gastric cancer. In our work on viral pathogenesis, the copy number of the EBV latent genome was found to correlate strongly with oncogenicity (Zuo et al., 2015). Emerging lines of evidence demonstrate the increasing functions and roles that exosomes have in the EBV tumorigenic process. Latent membrane protein 1 (LMP1) is the oncoprotein of EBV and is considered a key modulator in the pathogenesis of NPC. LMP1 causes the disfunction of signal transduction pathways such as NF-KB, STAT, and JAK3 (Zheng et al., 2007). Elevated telomerase activity is ubiquitous among individuals with cancers. LMP1 modulates telomerase activity through the p16INK4A/Rb/E2F1 and JNK signaling pathways in NPC (Ding et al., 2010). Moreover, LMP1 can also upregulate MDM2 protein, which is a cellular pro-oncogene that is frequently abnormally overexpressed in NPC, and lead to the initial vast accumulation of ubiquitinated MDM2 (Li et al., 2007). EBV traffics LMP1 into exosomes, altering the contents and function of exosomes. LMP1-modified exosomes promote the growth, migration, and invasion of cancer cells, and a tetraspanin protein, CD63, aids in the incorporation of LMP1 into exosomes. Moreover, CD63 is a critical partner of LMP1 to enhance exosome production and limit the activation of downstream signaling pathways such as NF-κB (Hurwitz et al., 2016). In addition, EBV-infected cells influence recipient cells and the surrounding microenvironment through exosomal miRNAs, Fas ligand, EBERs and other non-coding RNAs (Ahmed et al., 2015; Dolcetti, 2015; Iwakiri, 2015; Baglio et al., 2016; Gallo et al., 2016; Lin et al., 2016; Yoon et al., 2016; Zuo et al., 2017). MiRNAs are regarded as essential mediators in a variety of processes by orchestrating distinct targets and molecular pathways, and they are involved in immunological activities such as congenital immunity and virus infection (Li et al., 2010; Jia et al., 2014). EBV encodes its own miRNA and dysregulates host cellular miRNAs (Zuo et al., 2017; Yu et al., 2012). The miRNAs upregulated by EBV in cells are easily packaged into exosomes. Because miRNAs are usually enriched in exosomes, exosomal miRNAs are potential biomarkers of cancers and other diseases. Our recent work also showed that EBV-dysregulated long noncoding RNAs and cellular proteins may be transmitted to neighboring or distant cells (will be published elsewhere). This is a notable viral pathogenesis mechanism. In short, viruses hijack host exosomal pathways, and virally modified exosomes may accelerate the immune evasion, proliferation, and spread of the viruses (Meckes, 2015). Regarding virus-associated carcinoma, changes in exosome biology may contribute to tumor formation, development, metastasis, and invasion. Exosomes associated with EBV might be another possible approach to halt the progression of cancer besides the method of chemical agents like lactoferrin (Zheng et al., 2012).

KSHV is one of the multiplex carcinogenic viruses that are able to induce tumors in immunocompromised individuals. KSHV participates in the progression of Kaposi's sarcoma, HIV in immune-suppressed patients, and other pathogens (Thakker and Verma, 2016). KSHV establishes lifetime persistence and exhibits two different life cycles: a prolonged latent state and a short productive or lytic life cycle. In the former period, the virus integrates its episome into the host chromosome and is replicated during cell division. Latency-associated nuclear antigen (LANA), a multifunctional nuclear protein of KSHV, is expressed during latency. Pravinkumar Purushothaman proved that LANA has the capacity to modulate a diverse array of infections (Purushothaman et al., 2016). There are few available models for KSHV, but in a mouse model of KSHV and in patients with KSHV-associated malignancies, exosomal miRNAs of the host and those encoded by KSHV, including the miR-17-92 cluster, were successfully detected. Furthermore, the characterization and analysis of exosomal miRNAs, their targets, and endothelial cells revealed that exosomes containing a wide variety of miRNAs play a functional biological role in signaling pathways linked to the pathogenesis of KSHV and its phenotypic characteristics (Chugh et al., 2013; Zhang et al., 2015a). Hence, distinct exosomal miRNAs are expected to represent a portion of the paracrine signaling mechanism, which is the hallmark of KSHV tumorigenesis. Aside from miRNAs, gamma interferon-inducible protein 16 (IFI16), a nuclear pathogen sensor, and cleaved interleukin-1β (IL-1β), derived from the exosomes of B lymphoma cells, can be detected (Chugh et al., 2013). KSHV was found to utilize a strategy that disrupts the innate defense against inflammation. Indeed, due to the complexity of viral attachment and entry, KSHV enters cells by dynamin-dependent clathrin-mediated endocytosis and dynamin-independent micropinocytosis. It was revealed that irregular cup-shaped endocytic vesicles are required for successful infection (Veettil et al., 2014). However, no other prominent study on exosomes related to KSHV has been published to date.

## CONCLUSIONS

Herpesviruses are remarkable pathogens that efficiently utilize host components and resources to evade host immunity and achieve proliferation and egress. Although

Table 1. Exosomal molecules utilized by herpesviruses during infection

| Sub-families | Classification | Molecules related to exosomes |
|---|---|---|
| Alpha herpesvirus | HSV-1 | STING, miR-H28, miR-H29, ESCRT-III complex, Vps4, ALIX, TSG101, gB, DR, CD63 |
| | HSV-2 | none at present |
| | VZV | none at present |
| Beta herpesvirus | HCMV | TGN 46 and endosomes markers (annexin I, CD63, endosomal markers early endosomal antigen 1, transferrin receptor, and the cation-independent mannose 6-phosphate receptor) |
| | HHV-6 | CD63, MHC class I molecules, gB |
| | HHV-7 | none at present |
| Gamma herpesvirus | EBV | LMP1, CD63, miRNAs, Fas ligand, EBERs, and other non-coding RNAs, NF-κB, IFN, integrins, and actin |
| | KSHV | IFI16, IL-1β |

the mechanism related to EVs or exosomes utilized by herpesviruses has not been completely elucidated, available evidence suggests an elaborate process by which herpesviruses promote the interplay between infected and recipient cells. Herpesviruses inject genome expression products of their own and usurp cellular signaling molecules in exosome cargoes (Table 1). Viruses such as HSV-1 can even be packaged into MVBs, which have a similar size as EVs. These exosomal components function in downstream signaling. Therefore, exosomes not only can serve as a biomarker for the diagnoses of various diseases, including virus infections, but also could be targets used for the prediction and therapy of tumors or related diseases.

With the increasing number of studies on exosomes in virus infections, many issues need to be explored, including how the host exosomal proteins react to the virus infection, the molecular mechanism by which exosomes affect the envelopment and assembly of virions, and how herpesviruses assemble and egress with exosomes. The exploration of these questions will lay a better foundation for the development of vaccines and help obtain a deeper understanding of the pathogenesis of herpesvirus infection.

## ABBREVIATIONS

HHVs, human herpesviruses; HSV-1, herpes simplex virus 1; HSV2, herpes simplex virus 2; VZV, varicella-zoster virus; HCMV, human cytomegalovirus; EBV, Epstein-Barr virus; KSHV, Kaposi's sarcoma–associated herpesvirus; STING, the stimulator of IFN genes; Vps4, an enzyme required for producing luminal vesicles; MVB, multivesicular body; ESCRTs, the function of endosomal sorting complexes required for transport; ALIX and TSG101, two dominant-negative proteins; gB, HSV-1 encoded glycoprotein B; DR, HLA-DR; CD63, the late endosomal marker; EC, endothelial cells; TGN, the trans-Golgi network; U24, a protein of HHV-6; PEL, primary effusion lymphoma; NPC, nasopharyngeal carcinoma; LMP1, latent membrane protein 1; MDM2, a cellular pro-oncogene protein; LANA, Latency-associated nuclear antigen of KSHV; IFI16, gamma interferon-inducible protein 16; IL-1β, interleukin-1β.

## ACKNOWLEDGMENTS

This work was supported by the National Natural Science Foundations of China (81372139, 31670171, 81728011), the National Key Research and Development Program of China (2017YFC1200204), the Hunan Provincial Natural Science Foundation of China (2015JJ2149).

## COMPLIANCE WITH ETHICS GUIDELINES

The authors declare that they have no conflict of interest. This article does not contain any studies with human or animal subjects performed by any of the authors.

## OPEN ACCESS

This article is distributed under the terms of the Creative Commons Attribution 4.0 International License (https://creativecommons.org/licenses/by/4.0/), which permits unrestricted use, distribution, and reproduction in any medium, provided you give appropriate credit to the original author(s) and the source, provide a link to the Creative Commons license, and indicate if changes were made.

## References

[CR1] Agut H, Bonnafous P, Gautheretdejean A (2015). Laboratory and clinical aspects of human herpesvirus
6 infections. Clin Microbiol Rev.

[CR2] Ahmed W, Philip PS, Attoub S, Khan G (2015). Epstein-Barr virus infected cells release Fas-ligand
in exosomal fractions and induce apoptosis in recipient cells via the
extrinsic pathway. J Gen Virol.

[CR3] Akers JC, Gonda D, Kim R, Carter BS, Chen CC (2013). Biogenesis of extracellular vesicles (EV): exosomes,
microvesicles, retrovirus-like vesicles, and apoptotic bodies. J Neurooncol.

[CR4] Akinyi B, Odhiambo C, Otieno F, Inzaule S, Oswago S, Kerubo E, Ndivo R, Zeh C (2017). Prevalence, incidence and correlates of HSV-2
infection in an HIV incidence adolescent and adult cohort study in western
Kenya. PloS One.

[CR5] Anderson MR, Kashanchi F, Jacobson S (2016). Exosomes in Viral Disease. Neurotherapeutics.

[CR6] Arnold N, Messaoudi I (2017). Simian varicella virus causes robust transcriptional
changes in T cells that support viral replication. Virus Res.

[CR7] Baglio SR, van Eijndhoven MA, Koppers-Lalic D, Berenguer J, Lougheed SM, Gibbs S, Léveillé N, Rinkel RN, Hopmans ES, Swaminathan S (2016). Sensing of latent EBV infection through exosomal
transfer of 5’pppRNA. Proc Natl Acad Sci U S A.

[CR8] Calistri A, Sette P, Salata C, Cancellotti E, Forghieri C, Comin A, Göttlinger H, Campadellifiume G, Palù G, Parolin C (2007). Intracellular Trafficking and Maturation of Herpes
Simplex Virus Type 1 gB and Virus Egress Require Functional Biogenesis of
Multivesicular Bodies. J Virol.

[CR9] Cepeda V, Esteban M, Fraileramos A (2010). Human cytomegalovirus final envelopment on membranes
containing both trans-Golgi network and endosomal markers. Cell Microbiol.

[CR10] Chan T, Barra NG, Lee AJ, Ashkar AA (2011). Innate and adaptive immunity against herpes simplex
virus type 2 in the genital mucosa. J Reprod Immunol.

[CR11] Choi UY, Park A, Jung JU (2017). Double the Trouble When Herpesviruses Join
Hands. Cell Host Microbe.

[CR12] Chugh PE, Sin SH, Ozgur S, Henry DH, Menezes P, Griffith J, Eron JJ, Damania B, Dittmer DP (2013). Systemically Circulat-ing Viral and Tumor-Derived
MicroRNAs in KSHV-Associated Malignancies. PloS Pathog.

[CR13] Crump CM, Yates C, Minson T (2007). Herpes Simplex Virus Type 1 Cytoplasmic Envelopment
Requires Functional Vps4. J Virol.

[CR14] Ding L, Li L, Yang J, Zhou S, Li W, Tang M, Shi Y, Yi W, Cao Y (2010). Latent membrane protein 1 encoded by Epstein-Barr
virus induces telomerase activity via p16INK4A/Rb/E2F1 and JNK signaling
pathways. J Med Virol.

[CR15] Dolcetti R (2015). Cross-talk between Epstein-Barr virus and
microenvironment in the pathogenesis of lymphomas. Semin Cancer Biol.

[CR16] Dreyfus DH (2013). Herpesviruses and the microbiome. J Allergy Clin Immunol.

[CR17] Duijvesz D, Luider T, Bangma CH, Jenster G (2011). Exosomes as biomarker treasure chests for prostate
cancer. Eur Urol.

[CR18] Fraile-Ramos A, Pelchen-Matthews A, Risco C, Rejas MT, Emery VC, Hassan-Walker AF, Esteban M, Marsh M (2007). The ESCRT machinery is not required for human
cytomegalovirus envelopment. Cell Microbiol.

[CR19] Gallo A, Vella S, Miele M, Timoneri F, Di BM, Bosi S, Sciveres M, Conaldi PG (2016). Global profiling of viral and cellular noncoding
RNAs in Epstein-Barr virus-induced lymphoblastoid cell lines and released
exosome cargos. Cancer Lett.

[CR20] Han Z, Liu X, Chen X, Zhou X, Du T, Roizman B, Zhou G (2016). miR-H28 and miR-H29 expressed late in productive
infection are exported and restrict HSV-1 replication and spread in
recipient cells. Proc Natl Acad Sci U S A.

[CR21] Hancock MH, Skalsky RL (2017). Roles of Non-coding RNAs During Herpesvirus Infection.

[CR22] Hogue IB, Scherer J, Enquist LW (2016). Exocytosis of Alphaherpesvirus Virions, Light
Particles, and Glycoproteins Uses Constitutive Secretory
Mechanisms. Mbio.

[CR23] Hudson AW (2014). Roseoloviruses and their modulation of host
defenses. Curr Opin Virol.

[CR24] Hurley JH (2015). ESCRTs are everywhere. EMBO J.

[CR25] Hurwitz SN, Nkosi D, Conlon MM, York SB, Liu X, Tremblay DC, Meckes DG (2016). CD63 regulates Epstein-Barr virus LMP1 exosomal
packaging, enhancement of vesicle production, and non-canonical NF-kB
signaling. J Virol.

[CR26] Iwakiri D (2015). Multifunctional non-coding Epstein-Barr virus
encoded RNAs (EBERs) contribute to viral pathogenesis. Virus Res.

[CR27] Jia S, Zhai H, Zhao M (2014). MicroRNAs regulate immune system via multiple
targets. Discov Med.

[CR28] Meckes D Jr (2015). Exosomal Communication Goes Viral. J Virol.

[CR29] Meckes D J, Gunawardena HP, Dekroon RM, Heaton PR, Edwards RH, Ozgur S, Griffith JD, Damania B, Raab-Traub N (2013). Modulation of B-cell exosome proteins by gamma
herpesvirus infection. Proc Natl Acad Sci U S A.

[CR30] Kalamvoki M, Deschamps T (2016). Extracellular vesicles during Herpes Simplex Virus
type 1 infection: an inquire. Virol J.

[CR31] Kalamvoki M, Du T, Roizman B (2014). Cells infected with herpes simplex virus 1 export to
uninfected cells exosomes containing STING, viral mRNAs, and
microRNAs. Proc Natl Acad Sci U S A.

[CR32] Knipe DM, Raja P, Lee J (2017). Viral gene products actively promote latent
infection by epigenetic silencing mechanisms. Curr Opin Infect Dis.

[CR33] Kurapati S, Sadaoka T, Rajbhandari L, Jagdish B, Shukla P, Kim YJ, Lee G, Cohen JI, Venkatesan A (2017). Role of JNK pathway in varicella-zoster virus lytic
infection and reactivation. J Virol.

[CR34] Lee AJ, Ashkar AA (2012). Herpes simplex virus-2 in the genital mucosa:
insights into the mucosal host response and vaccine
development. Curr Opin Infect Dis.

[CR35] Lee Y, El AS, Wood MJ (2012). Exosomes and microvesicles: extracellular vesicles
for genetic information transfer and gene therapy. Hum Mol Genet.

[CR36] Li L, Chen XP, Li YJ (2010). MicroRNA-146a and human disease. Scand J Immunol.

[CR37] Li L, Li Z, Zhou S, Xiao L, Guo L, Tao Y, Tang M, Shi Y, Li W, Yi W (2007). Ubiquitination of MDM2 modulated by Epstein-Barr
virus encoded latent membrane protein 1. Virus Res.

[CR38] Lin Z, Swan K, Zhang X, Cao S, Brett Z, Drury S, Strong MJ, Fewell C, Puetter A, Wang X (2016). Secreted Oral Epithelial Cell Membrane Vesicles
Induce Epstein-Barr Virus Reactivation in Latently Infected B
Cells. J Virol.

[CR39] Lo AKF, Dawson CW, Young LS, Lo KW (2017). The role of Metabolic Reprogramming in
γ-Herpesvirus-associated Oncogenesis. Int J Cancer.

[CR40] Miettinen JJ, Matikainen S, Nyman TA (2012). Global Secretome Characterization of Herpes Simplex
Virus 1-Infected Human Primary Macrophages. J Virol.

[CR41] Mori Y, Koike M, Moriishi E, Kawabata A, Tang H, Oyaizu H, Uchiyama Y, Yamanishi K (2008). Human herpesvirus-6 induces MVB formation, and virus
egress occurs by an exosomal release pathway. Traffic.

[CR42] Olsson J, Kok E, Adolfsson R, Lövheim H, Elgh F (2017). Herpes virus seroepidemiology in the adult Swedish
population. Immun Ageing.

[CR43] Ota M, Serada S, Naka T, Mori Y (2014). MHC class I molecules are incorporated into human
herpesvirus-6 viral particles and released into the extracellular
environment. Microbiol Immunol.

[CR44] Parra M, Alcala A, Amoros C, Baeza A, Galiana A, Tarragó D G-Q M, Sánchez-Hellín V (2017). Encephalitis associated with human herpesvirus-7
infection in an immunocompetent adult. Virol J.

[CR45] Pawliczek T, Crump CM (2009). Herpes Simplex Virus Type 1 Production Requires a
Functional ESCRT-III Complex but Is Independent of TSG101 and ALIX
Expression. J Virol.

[CR46] Pegtel DM (2013). Oncogenic herpesviruses sending mixed
signals. Proc Natl Acad Sci U S A.

[CR47] Purushothaman P, Dabral P, Gupta N, Sarkar R, Verma SC (2016). KSHV Genome Replication and
Maintenance. Front Microbiol.

[CR48] Riva N, Franconi I, Meschiari M, Franceschini E, Puzzolante C, Cuomo G, Bianchi A, Cavalleri F, Genovese M, Mussini C (2017). Acute human herpes virus 7 (HHV-7) encephalitis in
an immunocompetent adult patient: a case report and review of
literature. Infection.

[CR49] Sotelo JR, Porter KR (1959). An Electron Microscope Study of the Rat
Ovum. J Biophys Biochem Cytol.

[CR50] Sullivan BM, Coscoy L (2010). The U24 protein from human herpesvirus 6 and 7
affects endocytic recycling. J Virol.

[CR51] Szatanek R, Bajkrzyworzeka M, Zimoch J, Lekka M, Siedlar M, Baran J (2017). The Methods of Choice for Extracellular Vesicles
(EVs) Characterization. Int J Mol Sci.

[CR52] Tandon R, Aucoin DP, Mocarski ES (2009). Human Cytomegalovirus Exploits ESCRT Machinery in
the Process of Virion Maturation. J Virol.

[CR53] Temme S, Eis-Hübinger AM, Mclellan AD, Koch N (2010). The herpes simplex virus-1 encoded glycoprotein B
diverts HLADR into the exosome pathway. J Immunol.

[CR54] Thakker S, Verma SC (2016). Co-infections and Pathogenesis of KSHV-Associated
Malignancies. Front Microbiol.

[CR55] Tkach M, Théry C (2016). Communication by Extracellular Vesicles: Where We
Are and Where We Need to Go. Cell.

[CR56] van Diemen FR, Lebbink RJ (2016). CRISPR/Cas9, a powerful tool to target human
herpesviruses. Cellular Microbiology.

[CR57] Veettil MV, Bandyopadhyay C, Dutta D, Chandran B (2014). Interaction of KSHV with host cell surface receptors
and cell entry. Viruses.

[CR58] Walker JD, Maier CL, Pober JS (2009). Cytomegalovirus-infected human endothelial cells can
stimulate allogeneic CD4+ memory T cells by releasing antigenic
exosomes. J Immunol.

[CR59] Wang J, Sun X, Zhao J, Yang Y, Cai X, Xu J, Cao P (2017). Exosomes: A Novel Strategy for Treatment and
Prevention of Diseases. Front Pharmacol.

[CR60] Yoon C, Kim J, Park G, Kim S, Kim D, Hur DY, Kim B, Kim YS (2016). Delivery of miR-155 to retinal pigment epithelial
cells mediated by Burkitt’s lymphoma exosomes. Tumor Biol.

[CR61] Zhang J, Zhu L, Lu X, Feldman ER, Keyes LR, Wang Y, Fan H, Feng H, Xia Z, Sun J (2015). Recombinant Murine Gamma Herpesvirus 68 Carrying
KSHV G Protein-Coupled Receptor Induces Angiogenic Lesions in
Mice. PloS Pathog.

[CR62] Zhang X, Yuan X, Shi H, Wu L, Qian H, Xu W (2015). Exosomes in cancer: small particle, big
player. J Hematol Oncol.

[CR63] Zheng H, Li L, Hu D, Deng X, Cao Y (2007). Role of Epstein-Barr Virus Encoded Latent Membrane
Protein 1 in the Carcinogenesis of Nasopharyngeal Carcinoma. Cell Mol Immunol.

[CR64] Zheng Y, Zhang W, Ye Q, Zhou Y, Xiong W, He W, Deng M, Zhou M, Guo X, Chen P (2012). Inhibition of Epstein-Barr Virus Infection by
Lactoferrin. J Innate Immun.

[CR65] Zhu Y, Yan Y, Guo J, Ying D, Ye L, Qiu J, Zeng Z, Wu X, Xing Y, Xiang L (2017). Ex vivo2D and 3D HSV-2 infection model using human
normal vaginal epithelial cells. Oncotarget.

[CR66] Zuo L, Yu H, Liu L, Tang Y, Wu H, Jing Y, Zhu M, Du S, Lian Z, Li C (2015). The copy number of Epstein-Barr virus latent genome
correlates with the oncogenicity by the activation level of LMP1 and
NF-kB. Oncotarget.

[CR67] Zuo L, Yue W, Du S, Xin S, Zhang J, Liu L, Li G, Lu J (2017). An update: Epstein-Barr virus and immune evasion via
microRNA regulation. Virol Sin.

